# Ovarian cancer: epigenetics, drug resistance, and progression

**DOI:** 10.1186/s12935-021-02136-y

**Published:** 2021-08-17

**Authors:** Weiwei Xie, Huizhen Sun, Xiaoduan Li, Feikai Lin, Ziliang Wang, Xipeng Wang

**Affiliations:** 1 Department of Obstetrics and Gynecology, Shanghai Jiao Tong University School of Medicine Xinhua Hospital, 1665 Kongjiang Road, Yangpu District, Shanghai, China; 2 Department of Gynecology, Shanghai First Maternity and Infant Hospital, Tongji University School of Medicine, Shanghai, China

**Keywords:** Epigenetics, Ovarian cancer, MiRNA, LncRNA, DNA methylation, Histone modifications

## Abstract

Ovarian cancer (OC) is one of the most common malignant tumors in women. OC is associated with the activation of oncogenes, the inactivation of tumor suppressor genes, and the activation of abnormal cell signaling pathways. Moreover, epigenetic processes have been found to play an important role in OC tumorigenesis. Epigenetic processes do not change DNA sequences but regulate gene expression through DNA methylation, histone modification, and non-coding RNA. This review comprehensively considers the importance of epigenetics in OC, with a focus on microRNA and long non-coding RNA. These types of RNA are promising molecular markers and therapeutic targets that may support precision medicine in OC. DNA methylation inhibitors and histone deacetylase inhibitors may be useful for such targeting, with a possible novel approach combining these two therapies. Currently, the clinical application of such epigenetic approaches is limited by multiple obstacles, including the heterogeneity of OC, insufficient sample sizes in reported studies, and non-optimized methods for detecting potential tumor markers. Nonetheless, the application of epigenetic approaches to OC patient diagnosis, treatment, and prognosis is a promising area for future clinical investigation.

## Background

Ovarian cancer (OC) is one of the deadliest, most malignant gynecological tumors. Due to its insidious onset, most patients have no specific manifestations or symptoms during the early stages of the disease. The lack of sensitive and efficient clinical screening methodology results in most diagnoses occurring at an advanced stage. Based on the latest statistics from the American Cancer Society, there are approximately 20,000 new cases of OC annually, accounting for 5% of all female malignant tumors, with a death rate of 62% [1]. The incidence of OC is increasing not only in Western countries, but also in Asian countries. Approximately 70% of patients with OC are diagnosed with advanced disease when the tumor has spread outside of the pelvis and to distant metastatic sites, which cannot be completely removed by surgery. The 5-year survival rate is 20–30%. The overall OC survival rate could be improved by the identification of specific biomarkers for early diagnosis. This type of discovery would represent a revolutionary breakthrough in OC research.

At present, the pathogenesis and specific etiology of OC are unclear. OC may be due to a combination of genetics, reproductive hormone levels, and behavior. The number of ovulations in a woman's lifetime is proportional to the risk for OC [2]. Among women who are not pregnant, menarche and late menopause result in increased ovulation and are high-risk factors for OC. Protective factors, such as pregnancy, term delivery, lactation, oral contraceptives, and tubal ligation, reduce the occurrence of OC [2]. Genetic factors contribute to approximately 10% of epithelial ovarian cancer (EOC) [2] and are usually characterized by the autosomal dominant inheritance of *BRCA1* or *BRCA2* genetic mutations [3]. The loss of function in genes encoding BRCA proteins results in the instability of tumor suppressors.

OC was initially thought to originate in the ovaries. With molecular biological analysis, the origin of OC has become controversial [4]. Currently, OC is believed to have three possible origins: ovarian surface epithelium (OSE), fallopian tube, or ectopic endometrial tissue [4, 5]. These tissues have the same embryological origin. According to the cell origin and histological features, OC is classified into epithelial, sex-cord stromal, germ cell and mixed-cell subtypes [6]. EOC is the most common cause of mortality in women with gynecologic tumors, accounting for 85–90% of all ovarian malignancies [7]. There are four main histologic subtypes of EOC: serous, clear-cell, mucinous, and endometrioid [8]. Fallopian tube cells may be the precursors of the most high-grade serous ovarian cancers (HGSOC) [9], and endometriotic cells may be the precursors of clear-cell and endometrioid tumors [10]. Its heterogeneity is believed to be the main reason for treatment failure and tumor drug resistance [11]. Tumors originating from different anatomical sites may be a possible cause of tumor heterogeneity [4].

Although each subtype has its own molecular and clinical characteristics, treatment for all epithelial ovarian subtypes remains similar, including de-bulking surgery and platinum-based chemotherapy. Regardless of the tissue type, platinum-based chemotherapy is the main treatment of choice for advanced EOC, typically carboplatin combined with paclitaxel [12]. For decades, intrinsic or acquired resistance to chemotherapy in most patients has inevitably posed a major barrier to the successful treatment of OC. Epigenetic drugs comprise a new generation of anticancer drugs that have unique interactions with tumor cells and associated microenvironments. These drugs may be used alone or in combination with classic chemotherapy. The key prognostic factor for OC is the resistance of the patient’s tumor to chemotherapy, especially platinum-based drugs. Epigenetic drugs combined with paclitaxel and platinum are more effective than chemotherapy alone [13].

## Epigenetics

**Fig. 1:**
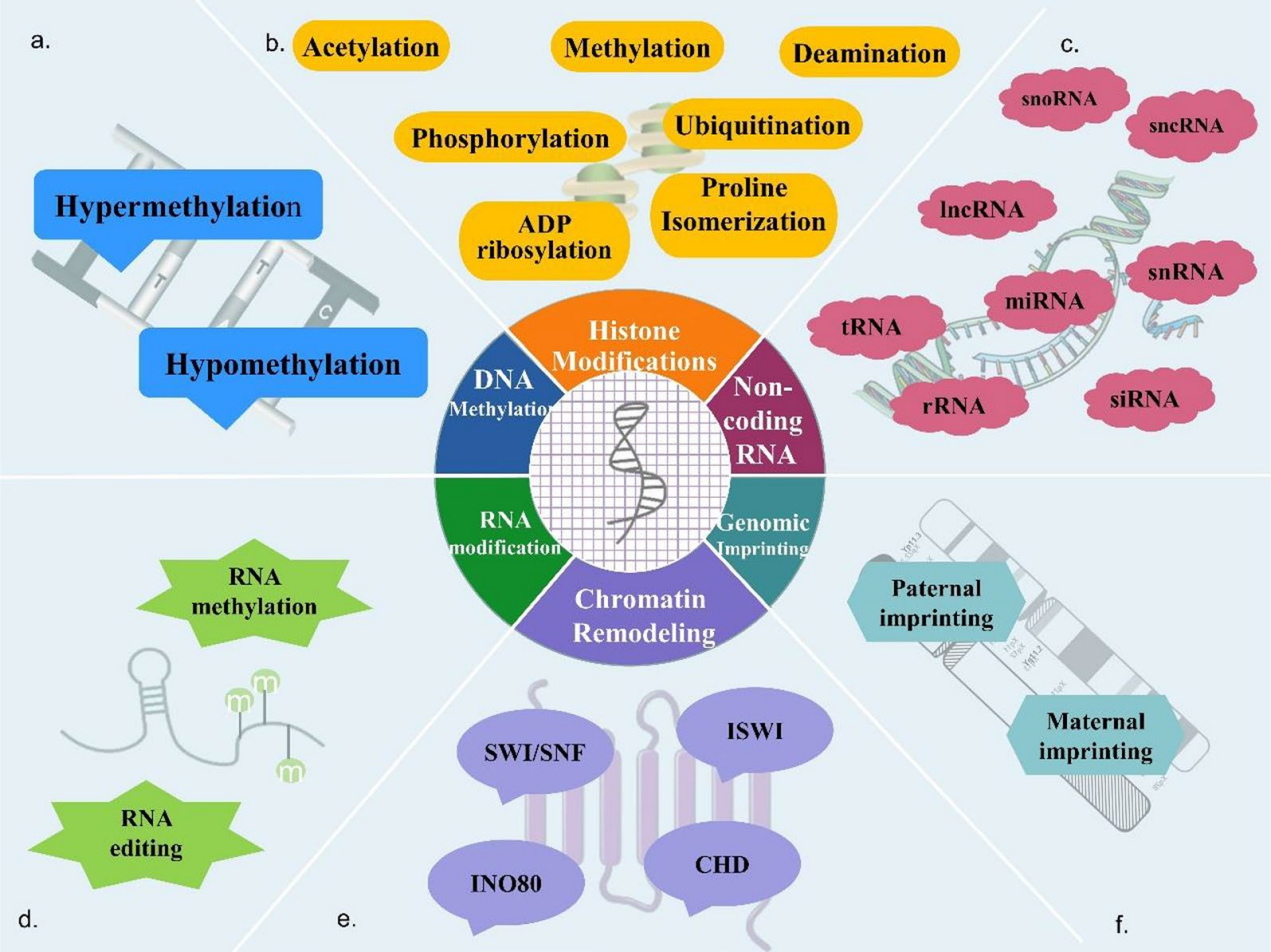
Epigenetic processes include DNA methylation, histone modification, non-coding RNA, RNA modification, chromatin remodeling, and genomic imprinting. **a** DNA methylation is divided into two categories: the hypermethylation of CpG islands and global hypomethylation. **b** Histone post-translational modifications, including methylation, acetylation, phosphorylation, deamination, ubiquitination, ADP‐ribosylation and proline isomerization. **c** Non-coding RNA including miRNA, lncRNA, rRNA, tRNA, snRNA, snoRNA, sncRNA, siRNA, etc., act in the nucleus or cytoplasm. **d** Post-transcriptional modification of RNA through RNA editing and RNA methylation. **e** Chromatin-remodeling complexes are grouped into four major families: SWI/SNF, INO80, ISWI, and CHD. **f** Genomic imprinting is an epigenetic process that mainly includes maternal imprinting and paternal imprinting

Without changing the DNA sequence, epigenetic processes influence the expression and function of genes, which may result in heritable phenotypes [14] (Fig. 1). Epigenetics serves as an adjunct to classical genetics, and epigenetic modifications are significantly influenced by changes in the internal and external environment, playing an important regulatory role in the transgenerational inheritance of acquired traits, fate of stem cells, and occurrence of cancer [15]. This article reviews recent evidence on OC, including occurrence and development, chemotherapy resistance, and the influence of epigenetic processes mediated by non-coding RNA (ncRNA), DNA methylation, and histone modification.

In the human genome, 95% of the DNA sequence does not encode proteins. The types of ncRNAs include ribosomal RNA (rRNA), transfer RNA (tRNA), microRNA (miRNA), long non-coding RNA (lncRNA), small nuclear RNA (snRNA), small nucleolar RNA (snoRNA), RNA interference (RNAi), small non-coding RNA (sncRNA), and short interfering RNA (siRNA) [16]. The importance of ncRNAs was not widely recognized until recently. Although ncRNAs cannot encode proteins, they have specific biological functions, such as the processing and modification of RNA, stabilization of mRNA, regulation of cell translation levels, transport of proteins, and chromatin structural modification [17].

**Fig. 2:**
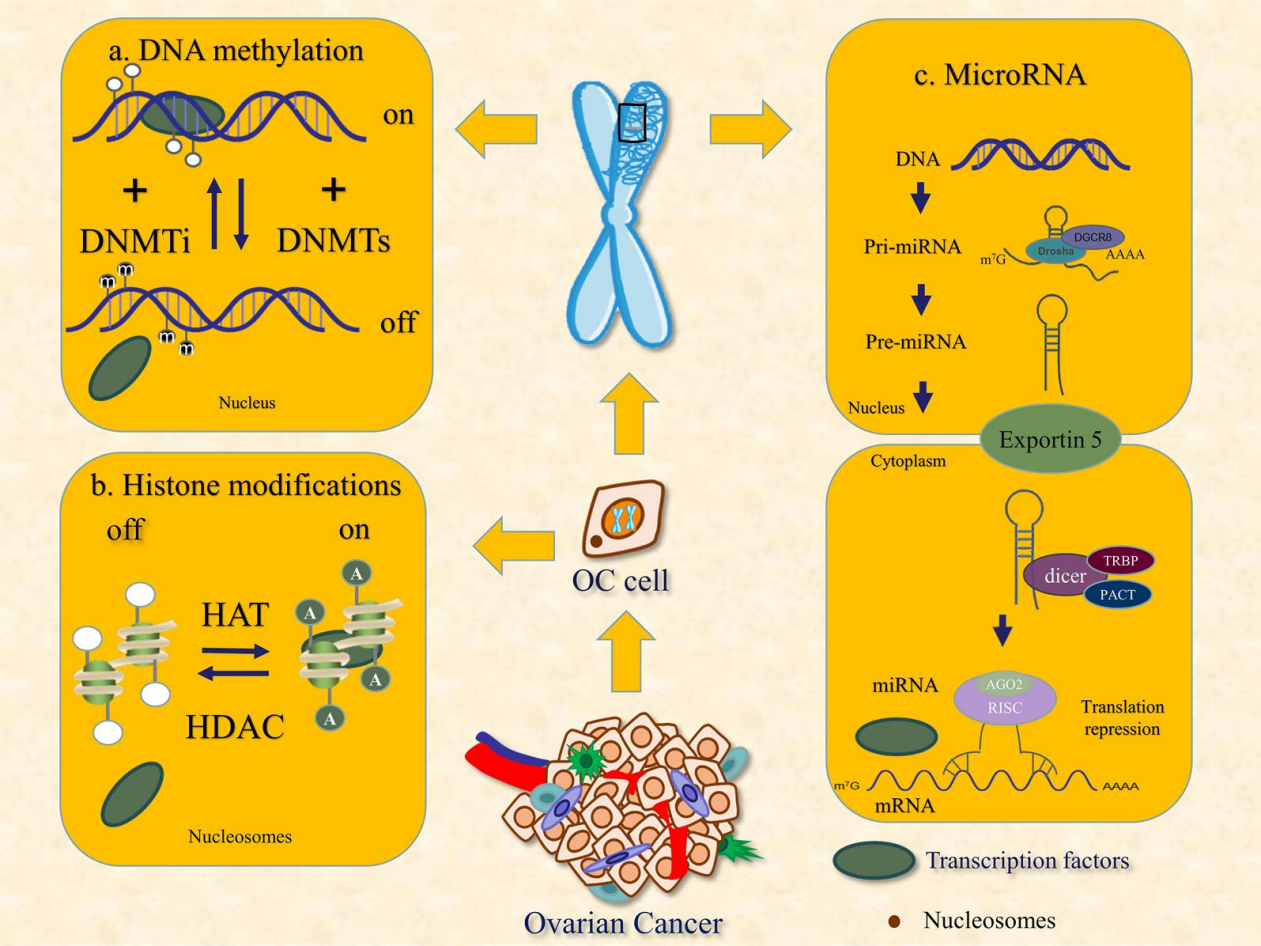
Classic molecular mechanisms of DNA methylation, histone modification, and  miRNA. **a** Genes are silenced by hypermethylation, which is catalyzed by DNA methyltransferases (DNMTs). Genes are expressed when DNA is demethylated, which is catalyzed by DNA methylation inhibitors (DNMTis). **b** Histone acetyltransferases (HATs) and histone deacetylases (HDACs) maintain a reversible equilibrium state of histone acetylation. **c** MiRNAs are formed from precursor RNAs that are cleaved by the Drosha and Dicer enzymes. MiRNAs block gene expression by promoting mRNA degradation and by preventing protein translation

MiRNA is an endogenous non-coding small molecule RNA with a length of 19–25 base pairs (bp), which is formed from its precursor RNA after cleavage by the Drosha and Dicer enzymes. Mature miRNA hinders translation extension by binding to the 3'UTR of target mRNA or by degrading RNA by promoting the separation of ribosomes and mRNA, thereby inhibiting gene expression [18] (Fig. 2). Nearly half of miRNA genes are located at fragile sites or chromosomal fragments that are amplified or deleted in human cancers, suggesting that miRNA is closely related to cancer [19].

**Fig. 3:**
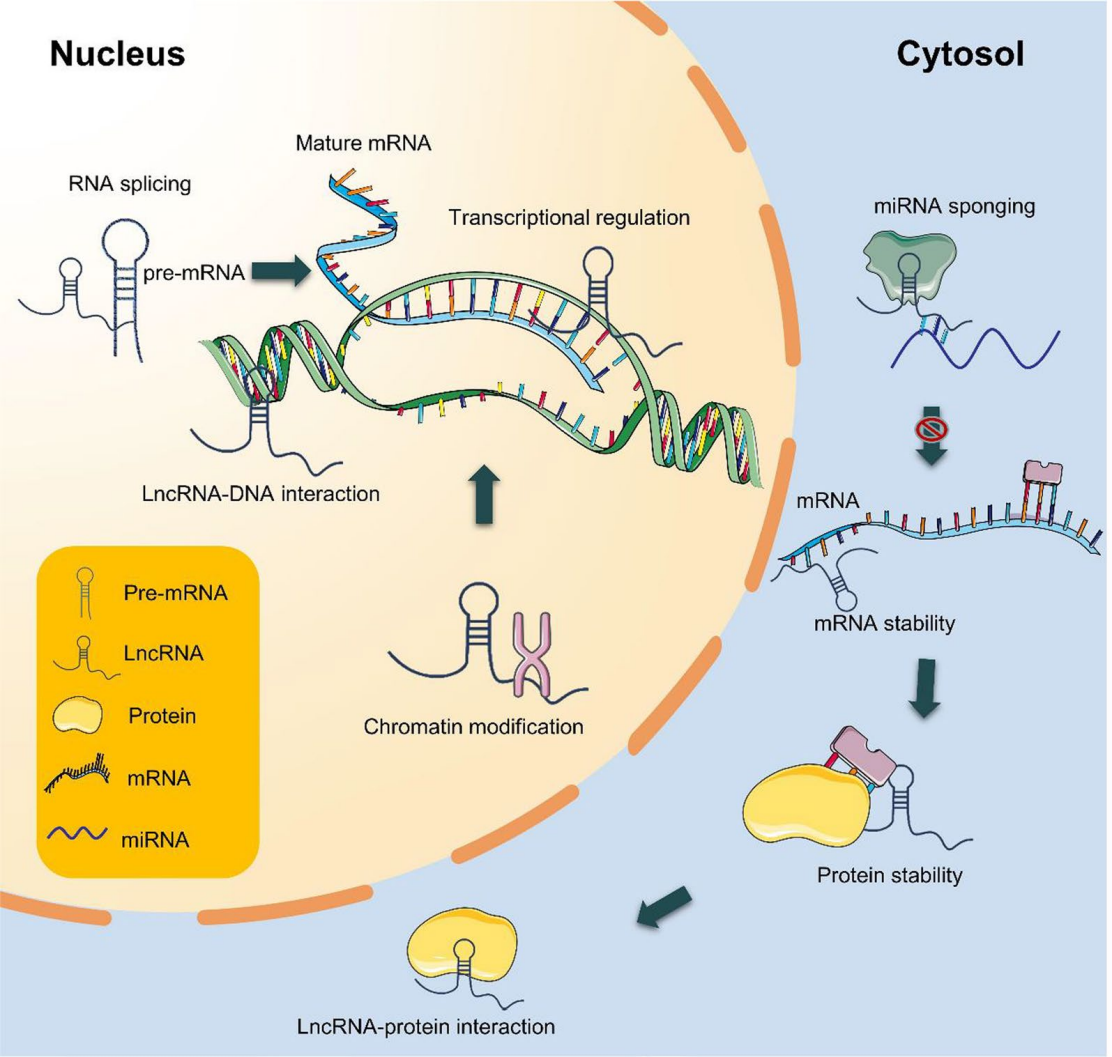
Schematic mechanisms of lncRNA in regulating gene expression. Nuclear lncRNAs modulate gene expression through chromatin modification, transcriptional regulation, RNA splicing and LncRNA–DNA interaction. In the cytoplasm, lncRNAs play a role in miRNA sponge formation, mRNA stability regulation and protein stability control

LncRNA is a subclass of ncRNA sequences with arbitrary lengths composed of more than 200 bp that were once considered "transcriptional noise" in genomic transcription. In recent years, through biotechnology and high-throughput sequencing, the abnormal expression of lncRNAs has been tightly associated with the biological behavior of tumors through epigenetic and post-transcriptional regulation [20] (Fig. 3). Based on their genomic positions relative to protein-coding genes, lncRNAs can be classified into five major categories: sense, antisense, pseudogenes, intergenic, intronic, and bidirectional promoters [21]. The most common epigenetic modifications of lncRNAs in tumors are imprinting loss or methylation changes (hypomethylation and hypermethylation). LncRNAs are involved in the regulation of imprinted gene networks. For example, as a transcriptional regulator, H19 regulates tumor growth by transfecting a gene imprinting network [22]. LncRNAs recruit chromatin epigenetic modification factors and change the looseness and tightness of chromatin to achieve chromatin remodeling, thereby regulating gene expression [23]. LncRNAs can recruit DNA methyltransferases, leading to either methylation or demethylation [24]. In addition, lncRNAs are involved in epithelial–mesenchymal transformation (EMT) [25] and cell stemness [26].

Interestingly, lncRNAs interact with miRNAs in multiple ways. For example, lncRNAs act as molecular sponges to bind miRNAs and inhibit their binding to mRNA [27]; as precursors of miRNAs, lncRNAs are cleaved by the Dicer enzyme to form mature miRNAs. LncRNAs bind to target miRNAs to promote their degradation, and competition occurs between the two molecules at the same mRNA site [28].

DNA methylation is the most commonly studied epigenetic modification of malignant tumors (Fig. 2). Expressed genes are generally not methylated. In many forms of cancer, tumor suppressor and DNA repair genes are often hypermethylated and silenced. The abnormal methylation of *CpG* islands (CpG-rich regions, 500–1000 bp in length with GC content exceeding 55%) can regulate the cell cycle, drug sensitivity, and tumor suppressor gene silencing. The aberrant methylation of DNA changes gene expression, which can promote damage and tumorigenesis [29]. It cannot be ignored that the abnormal methylation of other genomic loci, such as enhancers and repetitive elements, is also the main driving factor for tumor occurrence and development [30]. Furthermore, some evidence suggests that DNA hypomethylation associated with cancer may increase genomic instability [30]. However, promoter DNA methylation does not always act as a transcriptional silencing mechanism. It has been discovered that DNA hypomethylation promotes tumorigenesis through the transcriptional activation of oncogenes [31]. These findings suggest that epigenetics contributes to transcriptional regulation in a more dynamic and complex manner than previously believed.

After histone translation is complete, the amino terminus is covalently modified to regulate the expression of the corresponding gene. Changes in chromatin structure caused by covalent modification are known as ubiquitination, glycosylation, deamination, ADP ribosylation, and proline isomerization [32]. Acetylation and methylation are the two most important modifications that function by upregulating or downregulating gene expression, respectively [33]. Histone modification plays an important role in gene transcription, DNA damage repair, DNA replication, and chromosome condensation [34]. Histone acetyltransferases (HATs) and histone deacetylases (HDACs) maintain a reversible and dynamic equilibrium of histone acetylation (Fig. 2). Histone methylation occurs on lysine or arginine residues of histones H3 and H4. The methylation of histone H3 lysine 27 (H3K27) is related to the silencing of many genes, such as genes imprinted and inactivated on the X chromosome [35].

In addition, a series of epigenetic modifications have recently been discovered, such as acetylation modifications of RNA, glycation modifications and lactate modifications of histones, whose functions need further exploration. The polycombgroup of proteins (PcG) remodels chromatin and epigenetically silences genes [36].

## Application of epigenetics in OC

### NcRNA in OC

#### MiRNA

MiRNAs regulate the expression of oncogenes and tumor suppressor genes in OC through a complex circulatory network that controls tumor proliferation, apoptosis, invasion, metastasis, and immune escape [37]. Along with miRNA molecules involved in the pathogenesis of malignant tumors, essential components of miRNA processing (Dicer, Drosha, DGCR8, Argonaut, and TRBP) are also involved [38].

##### MiRNAs as biomarkers

After the miRNA expression profiles of 894 EOC samples were analyzed (the largest collection to date available), 35 miRNAs predicting the risk of progression or relapse were identified. Among them, 16 were associated with a better prognosis, and 19 with a worse prognosis [39]. Due to the enormous number of such reports, this article lists some examples (Table 1). MiRNA is abnormally expressed in different tissue types of OC and can also be detected in body fluids such as blood, ascites, and urine [40]. Due to effective detection in body fluids, high stability, and tissue-specific expression patterns, miRNAs have potential as novel biomarkers. MiR-34 induces the autophagy and apoptosis of tumor cells, regulates tumor proliferation, and targets notch-1, thereby inhibiting cell invasion in OC [41]. Serum miR-375 and miR-1307 are upregulated in OC and may be used to support diagnosis in combination with CA-125 [42]. The overexpression of miR-9 may promote the cell migration and invasion of OC by targeting E-cadherin and become a new potential marker to control the metastasis of OC [43].

**Table 1:** Expression of miRNA in OC

Subtype	Up-regulation	Down-regulation	Target/pathway	Reference
Serous	miR-9		E-cadherin	[43]
Epithelial		miR-219-5p	Twist/Wnt/β-catenin signaling pathway	[44]
Serous	miR-616		TIMP2	[130]
Hyaline cell		miR-424	doublecortin-like kinase 1	[131]
High-grade serous	miR-1290			[132]
Epithelial	miR-99a-5p		fibronectin and vitronectin	[133]
Epithelial	miR-1181, miR-4314		FOXP1 and GRWD1/IP6K1/NEGR1	[134]
Clear cell and endometriosis		miR-381	PIK3CA	[135]
Epithelial		miR-802	YWHAZ,	[136]
Epithelial		let-7g	c-Myc and cyclin-D2	[46]
Epithelial		miR-542-3p	CDK14	[137]

While some miRNAs have been suggested to be involved in the proliferation and invasion of OC, others may play opposing roles. MiRNA-219-5p inhibits the invasion, proliferation, and migration of EOC by targeting the Twist/Wnt/β-catenin signaling pathway, suggesting its potential role in the diagnosis and treatment of EOC [44]. By targeting multiple oncogenic genes, the classic Let-7 family of miRNAs has a tumor suppressor function. Its expression is downregulated in many cancer cells [45]. Let-7g overexpression induces a significant reduction in OC cancer cell growth. This effect leads to partial arrest of the G0/G1 cell cycle and significant downregulation of c-Myc and cyclin-D2 in OVCAR3 and HEY-A8 cells [46]. The architectural transcription factors HMGA2 and LIN28B and the RNA-binding protein IGF2BP1 form a self-promoting oncogenic "triangle" that adequately antagonizes the tumor inhibitory effects of the let-7 miRNA family [47]. The let-7 antagonistic triangle may be active in a wide range of cancers along with in OC. Impairing the potential of this triangle by targeting let-7 could be a new direction for the diagnosis of early OC. These findings clearly indicate that aberrant expression of miRNAs may serve as novel biomarkers for the diagnosis, prognosis and monitoring of OC.

##### MiRNAs as therapeutic targets

The differential expression of miRNAs is a double-edged sword in OC. Platinum and paclitaxel are two types of drugs that have been studied in detail to explore the effects of miRNAs on the sensitivity and resistance to chemotherapy in OC. The upregulation or downregulation of specific miRNAs has the potential to modulate the responsiveness of OC cells to chemotherapy (Table 2). Neoadjuvant chemotherapy (NACT) has been recognized as a reliable treatment strategy for patients with advanced EOC. The molecular mechanisms leading to platinum reaction in NACT settings have not been explored. Longitudinal analysis of miRNA expression profiles in HGSOC patients treated with NACT reveals that the expression levels of miR let-7G-5p, miR-199a-3p, miR-199a-5p, and miR181a-5p are independently associated with OS and PFS [48]. Moreover, the above-mentioned four miRNAs are correlated with Pt-based resistance and prognosis. Concomitant expression of P-Smad2 and miR181a-5p in surgical samples may be capable of confirming a poor outcome and little chance of response to Pt-based NACT.

**Table 2:** Application of miRNA to OC drug resistance

miRNA	Resistance against	Function	Target/Pathway	References
miR-708	Cisplatin	Inhibition of metastasis	IGF2BP1/Akt	[49]
miR-136	Paclitaxel	Inhibition of proliferation	Notch3	[51]
miR-744-5p	Carboplatin	Promotion of cell apoptosis	NFIX and HNRNPC	[138]
miR-98-5p	cisplatin	Promotion of drug resistance	miR-98-5p/Dicer1/miR-152	[58]
miR-1246	Paclitaxel	Promotion of tumor growth	Cav1/p-gp/M2-type macrophage axis	[53]
miR-142-5p	Cisplatin	Inhibition of drug resistance	XIAP, BIRC3, BCL2, BCL2L2, and MCL1(?)	[139]
miR-509-3p	Platinum	Enhance drug sensitivity	GOLPH3 and WLS	[140]
miR-34a	Cisplatin	Inhibition of proliferation	HDAC1	[50]
miR-338-3p	Cisplatin	Inhibition of proliferation, motility, and EMT	WNT2B	[141]
miR-1307	Paclitaxel	Affects cell cycle dynamics	CIC	[142]
miR-383-5p	Paclitaxel	Tumor suppressor	TRIM27	[52]
miR-206		Inhibition of proliferation and metastasis	c-Met/AKT/mTOR Signaling Pathway	[143]
miR-503-5p	Paclitaxel	Inhibition of tumor angiogenesis and growth	CD97-Mediated JAK2/STAT3 Pathway	[54]
miR-30a-5p		Inhibition of migration and invasion	SKP2, BCL9, and NOTCH1	[144]
miR-34a-5p	Cisplatin	Inhibition of proliferation and G1-phase cell cycle	PD-L1	[145]

MiR-708 increases the sensitivity of cisplatin-resistant cells through the IGF2BP1/Akt pathway [49]. MiR-34a downregulates HDAC1 expression while inhibiting proliferation and reducing resistance to cisplatin in OC cells [50]. MiR-136 re-sensitizes OC cells to paclitaxel by targeting the Notch-3 oncogene [51]. The expression of miR-383-5p is downregulated in OC, while the expression of TRIM 27 is upregulated [52]. MiR-383-5p inhibits cell proliferation and enhances paclitaxel chemosensitivity by suppressing TRIM27 expression. Interestingly, oncogenic miR-1246 has been found in OC, and its inhibitor has a significant sensitization effect on paclitaxel [53]. A new mechanism by which miR-503-5p induces metastasis in chemoresistant OC cells has recently been discovered [54]. MiR-503-5p inhibits the colony formation and metastasis of paclitaxel-resistant OC cells by inhibiting the CD97-mediated JAK2/STAT3 pathway. MiR-141/KLF12/Sp1/survivin, as a new signaling axis, can enhance the drug resistance of OC and may be a potential target for the treatment of metastatic OC [55]. In addition, miR-200c has been proposed as a potential circulating biomarker in OC to predict the outcome of bevacizumab combined with standard chemotherapy over standard chemotherapy alone [56].

As a tumor suppressor, let-7g may be used to inhibit tumor progression and resistance to cisplatin chemotherapy in EOC [46]. Snai1 is a major regulator of epithelial–mesenchymal transition (EMT). Interestingly, the expression of the tumor suppressor gene let-7 was upregulated in snail knockout cells. These findings suggest that the Snail/Let-7 axis may be an appealing target for HGSOC therapy [57]. Unlike traditional tumor suppressors, miR-98-5p, as a member of the let-7 family, shows the greatest inhibitory effect on Dicer1 and is significantly upregulated in cisplatin-resistant EOC cell lines [58]. MiR-98-5p promotes chemo-resistance to cisplatin through a novel miR-98-5p/DICER1/miR-152 pathway. These results may provide new predictive and prognostic ideas for OC and aid in the design of new miRNA-based therapeutic strategies.

MiRNA detection methods are continually improved. Ongoing research aims to establish a drug delivery system that reduces their local accumulation, systemic toxicity, and side effects. The use of porous anti-miRNA nanoparticles for OC therapy is a new form of targeted therapy [59]. However, it is not clear how each miRNA can be applied to overcome drug resistance. In conclusion, the discovery of miRNAs and their application in the pathophysiology of OC create unlimited possibilities for the transformation of miRNA scientific research into clinical applications.

#### LncRNA

Unlike miRNAs and other non-coding transcripts, lncRNAs are complex and large, and their ability to regulate genes in almost all transition states indicates their potential [60] (Table 3). Abnormal expression levels of these lncRNAs can be detected in body fluids and tumor tissues. In EOC, hundreds of lncRNAs are differentially expressed compared with benign and normal control tissues [61]. LncRNAs exhibit a significant association with disease-free survival (DFS) and overall survival (OS) clinical outcomes, both individually and as part of molecular signatures [62, 63]. By exploring and analyzing The Cancer Genome Atlas (TCGA) data, a 10-lncRNA prognostic signature can be used to assess the clinical outcomes of patients with HGSOC. Patients are classified into low-, medium-, and high-risk groups, with a significantly shortened OS and DFS in the high-risk group [63]. LncRNAs play roles in the pathogenesis and drug resistance to therapy in OC through various mechanisms, including aberrant lncRNA expression and single-nucleotide polymorphisms of functional lncRNAs. Recently, antisense and intergenic lncRNAs have been shown to regulate cell behavior in a variety of cancer types [64]. The expression of lncRNAs has been used to identify non-coding transcriptional markers associated with the prognosis of stage I EOC [65]. A signature composed of six different lncRNAs (*RUNX1-IT1*, *MALAT1*, *H19*, *HOTAIRM1*, *LOC100190986*, and *AL132709.8*) is significantly correlated with recurrence in OC [66]. Gene Ontology (GO) enrichment and Gene Set Enrichment Analysis (GSEA) identified five reliable lncRNAs (*LINC00664*, *LINC00667*, *LINC01139*, *LINC01419* and *LOC286437*) that are involved in multiple mechanisms of OC. The five lncRNAs are independent risk factors for OC recurrence [67].

**Table 3:** Molecular function of lncRNA in OC

LncRNA	Adjust	Resistance against	Function	Target/pathway
HOTAIR	↑	Cisplatin	Correlates with DNA damage response and senescence	NF-κB [70]; HOXA7 [146]; miR-200c [147]; P65, caspase 3/9 [148]; MMP9, MMP3, E-cadherin, vimentin, snail [69]; Wnt/β-catenin [149];
H19	↑	Cisplatin	Promotion of cell migration and invasion	IGF2 [79]; EZH2, P21, PTEN [150]; miR-370-3p [151]; let-7 [80]; miR-370-3p-TGF-beta [151];
MALAT1	↑	Cisplatin	Promotion of cell proliferation, migration, and invasion	Notch1 [76]; miR-200c [90]; PI3K/AKT pathway [77];
MEG3	↓	Cisplatin	Tumor suppressor	EMT [152]; miR-214 [86];
UCA1	↑	Paclitaxel Cisplatin	Apoptosis; drug effluent system	miR-143/FOSL2 pathway [153];
XIST	↑	Cisplatin	Promotion of cell proliferation, migration, and invasion	hsa-miR-214-3p [93];
BC200	↓	Carboplatin	Tumor suppressor	Inhibited cell proliferation and increased the sensitivity of OC cells to carboplatin [154];
LSINCT5	↑	Paclitaxel	Promotion of cell proliferation, migration, and invasion	CXCL12/CXCR4 [155];
DNM3OS	↑	Cisplatin	Promotion of cell proliferation, migration, and invasion	EMT [152];
NEAT1	↑	Paclitaxel	Promotion of cell proliferation and metastasis Inhibition of apoptosis	miR-383-3p [95]; ZEB1 [81];
ANRIL	↑	Cisplatin	Promotion of cell proliferation and cell cycle progression	let-7a/HMGA2 [83];
GAS5	↓	Platinum	Induction of apoptosis	cyclin D1, p21, APAF1 [85];

↓: Downregulated; ↑: Upregulated

##### HOTAIR

HOX antisense intergenic RNA (*HOTAIR*) was significantly increased in 44 OC tissues compared with 14 normal ovary tissues [68]. *HOTAIR* levels were positively correlated with the FIGO stage, histological grade of the tumor, lymph node metastasis and reduced OS and DFS [69]. In OC, *HOTAIR* is upregulated and positively correlated with the transcription factor nuclear factor kappa B (NF-κB) levels [70]. The NF-κB–HOTAIR axis drives a positive-feedback loop cascade in the DNA damage response and contributes to cell senescence and chemotherapy resistance in OC. The knockdown of *HOTAIR* can increase OC sensitivity to cisplatin by inhibiting cisplatin-induced autophagy [71]. The metastasis-promoting effect of *HOTAIR* is mediated by the regulation of the expression of many genes involved in EMT and cell metastasis, including matrix metalloproteinase 3(MMP3), MMP9, E-cadherin, vimentin and snail [69].

##### MALAT1

In the microarray analysis of lncRNAs, metastatic specific lung adenocarcinoma transcript 1 (*MALAT1*) was found to be significantly increased in OC tissues and cell lines [72, 73]. *MALAT1* upregulation promotes the proliferation, migration, invasion, and metastasis of OC cells in vivo [74, 75]. Survival analysis reveals that patients with increased *MALAT1* expression have a poorer DFS time [73]. *MALAT1* knockdown significantly reduced cisplatin resistance in OC by inhibiting the Notch1 signaling pathway [76]. *MALAT1* also promotes metastasis and proliferation through the PI3K/AKT pathway in EOC [77]. Both *HOTAIR* and *MALAT1* have been identified as potential therapeutic targets for restoring platinum sensitivity.

##### H19

The *H19* gene is the first identified imprinted lncRNA with maternal expression. Together with a neighboring gene, insulin-like growth factor 2 (IGF2), *H19* plays a key role in early pregnancy and normal menstrual cycles [78]. Recent studies have found that IGF2/H19 variants are significantly associated with genetic susceptibility to EOC in Han Chinese women [79]. As mentioned above, *H19* is associated with OS and DFS in OC [66]. Interestingly, these events can be modulated by inhibiting let-7 and subsequently enhancing the expression of the target genes HMGA2, c-Myc, and IGF2BP, which promote metastasis [80].

##### Other lncRNAs

High expression of nuclear paraspeckle assembly transcript 1 (*NEAT1*) has been observed in both OC cell lines and tumors. *NEAT1* promotes drug resistance to paclitaxel by upregulating ZEB1 expression by sponging miR-194 [81]. Compared with non-cancer tissues, the expression of *ANRIL* is significantly increased in EOC tissues, and increased *ANRIL* levels are associated with advanced FIGO stage, high histological grade, and poor OS [82]. Mechanistic investigations in vitro confirmed that silencing *ANRIL* promoted apoptosis and enhance the cisplatin sensitivity of OC cells by upregulating let-7a expression [83]. Furthermore, the upregulation of the lncRNAs long stress-induced non-coding transcript 5 (*LSINCT5*), colon cancer-associated transcript 2 (*CCAT2*), competing endogenous lncRNA 2 (*CERNA2*), *PVT1*, and urothelial cancer-associated 1 (*UCA1*) have also been implicated in cancer-promoting mechanisms of OC [84].

In contrast, some lncRNAs are downregulated in OC. Reduced expression of brain cytoplasmic RNA 200 (*BC200*) and growth arrest-specific 5 (*GAS5*) has been observed in OC cells and tissues. *GAS5* promotes OC tumorigenesis through its downstream effects on genes related to cell cycle progression, namely, P21, cyclin D1 and APAF1 [85]. LncRNA *MEG3* is down expressed in OC. *MEG3* upregulation can reduce the cisplatin resistance of OC cells by reducing EVS-mediated miR-214 [86]. In OC cells, lncRNA *GAS5* inhibits the cell cycle and promotes apoptosis by reducing cyclin D1, p21 and APAF1 levels [85]. However, to date, few lncRNAs have been identified that could play key roles in the treatment of OC.

#### LncRNA–miRNA interactions

As mentioned above, lncRNAs mainly act as sponges that bind miRNAs and inhibit their functions. As a tumor suppressor gene, miR-129 has been reported to inhibit the proliferation and invasion of lung cancer and breast cancer [87, 88]. LncRNA *SNHG12* is overexpressed in OC, and its expression level shows a positive association with tumor size and FIGO stage. As a molecular sponge of miR-129, *SNHG12* can directly bind to miR-129 and inhibit the function of miR-129, resulting in carcinogenesis [89]. *MALAT1* also acts as a sponge for miR-200c and inhibits tumor growth through miR-506-dependent iASPP [90, 91]. LncRNA *LINC01133* is downregulated in OC and acts as a negative regulator for miR-205, upregulating leucin-rich repeat kinase 2 (LRRK2) to inhibit OC development [92]. The lentivirus transfection of *XIST* into CAOV3 and OVCAR3 cell lines confirms that *XIST* can also directly act as a miRNA sponge to bind miR-214-3p and inhibit its expression, thereby inhibiting EOC development and increasing cisplatin chemosensitivity [93].

Furthermore, lncRNAs have a synergistic effect on miRNAs. *MEG3* mentioned above plays a role in targeting miR-214. *HOTAIR* promotes the proliferation and migration of OC cells via the miR-373 regulatory network [94]*. NEAT1* promotes the infiltration and metastasis of OC cells by regulating the miR-382-3p/ROCK 1 axis [95]. LncRNA-*TUSC7* is repressed in patients with OC, and reduced *TUSC7* promotes invasion, proliferation and migration of OC cells through the miR-616-5p/GSK3β/β-catenin pathway [96]. These studies continue to improve the understanding of ncRNAs in OC. However, the extent to which these interactions are functionally relevant in cells is still a matter of debate.

### DNA methylation and histone modification

The aberrant methylation of some oncogenes and tumor suppressor genes has been extensively investigated. The aberrant methylation of CpG islands in ovarian tumors is related to the regulation of the cell cycle, apoptosis, drug sensitivity, and the silencing of tumor suppressor genes. Demethylating agents can activate RNA transcription of silent endogenous retroviruses, stimulate antiviral interferon (IFN) signal transduction, and activate antitumor immune responses [97]. Thus, the regulation of gene expression by DNA methylation may play an important role in the gene competition between viruses and hosts.

The classic tumor suppressor gene, *BRCA1*, was discovered in 1994 on chromosome 17q12-21. The hypermethylation of *BRCA1* causes its expression to be decreased or deleted, inducing abnormal cell proliferation and affecting cell differentiation. The abnormal methylation of the dominant 5'UTR promoter leads to BRCA1 gene silencing, which is one of the causes of OC [98]. The abnormal methylation of *BRCA1* occurs in up to 15% in EOC [99], which is related to the initiation of OC and can therefore be used for targeted therapy. Poly-ADP ribose polymerase inhibitors (PARPis) are the first targeted drugs for OC [100]. PARPis prevent cells from repairing single-strand DNA damage, improve the 5-year survival of OC patients with *BRCA1* mutations, and increase the sensitivity to platinum drugs due to the disruption of DNA repair. The benefits of PARPis are not limited to *BRCA* mutation carriers but also extend to wild-type *BRCA* carriers [101]. In patients with advanced OC who received first-line standard treatment that included bevacizumab and maintenance olaparib (a PARPi), a significant progression-free survival benefit was observed [102]. Therefore, the abnormal methylation of *BRCA1* is closely related to the initiation of OC, targeted therapy, and prognosis.

Through DNA damage and cell cycle imbalance, the disruption of the histone methyltransferases EHMT1/2 (*GLP/G9A*) induces HGSOC cancer cell sensitivity to PARPis [103]. Ep-100 is a lytic peptide that specifically targets gonadotropin-releasing hormone receptors on cancer cells. Its combination with olaparib significantly increases the phosphorylation of histone H2AX in OC cells, exacerbating DNA damage [104]. Thus, the combination of ep-100 and olaparib is a promising treatment strategy. All-trans retinoic acid (ATRA), a targeted drug used to treat hematological malignancies, can inhibit cell proliferation in telomerase reverse transcriptase (TERT)-hypomethylated OC tissue types, and ATRA may be a new and effective individualized therapy [105]. Ubiquitin-specific protease 1 (USP1) stabilizes *SNAIL* by deubiquitination, thus promoting platinum resistance and OC cancer cell proliferation. The inhibition of USP1, in combination with platinum compounds, could be a successful strategy to improve platinum efficacy [106]. These studies demonstrate that there are other possible mechanisms in epigenetics that still need to be explored.

The antitumor efficacy of bromodomain and extra-terminal motif protein inhibitors (BETis) has been demonstrated in numerous types of cancers. It has been estimated that members of the Switch/Sucrose Non-Fermentable (SWI/SNF) chromatin remodeling complex (including SMARCA4, ARID1A and PBRM1 subunits) are mutated in approximately 20% of all tumor types [107]. Aggressive OCs lacking SMARCA4 and SMARCA2 may be highly sensitive to BETis [108]. BETis show the highest level of antitumor activity when both SMARCA4 and SMARCA2 are mutated or lost. ARID1A is mutated in more than 50% of ovarian clear-cell carcinomas [109]. BRD2, a member of the BET family, specifically inhibits the proliferation of ARID1A-mutated cell lines [110]. There is an unexpected and lethal interaction between BRD2 deletion and ARID1A mutation. BETis lead to a reduction in the expression of multiple SWI/SNF members and may be a novel method for the treatment of ARID1A-mutated ovarian clear-cell carcinomas. Furthermore, BETis may enhance DNA damage induced by PARPis through homologous recombination [111].

To date, antitumor epigenetic drugs that have been marketed mainly consist of four categories. Two of the most classic epigenetic therapies investigated are DNMTis and HDACis [112] (Table 4). Histone demethylase inhibitors and histone methyltransferase EZH2 inhibitors are other antitumor drugs that have received much attention in the field of epigenetics. EZH2, a member of Polycomb Repressor Complex 2 (PRC2), is commonly involved in transcriptional repression and overexpressed in OC. Although many ongoing clinical trials are currently using EZH2 inhibitors, no one is studying the use of EZH2 inhibitors in OC patients [113].

**Table 4:** Application of DNMTi and HDACi to OC treatment

Drugs	Function	Reference
DNMTi		
SGI-110	1. Drug sensitizer used in combination with cisplatin	[156]
	2. Enhances the immune recognition of tumor cells by regulating MHC class I and immunomodulatory molecules in EOC cells, and is superior to AZA or DAC	[157]
5-Aza-2’-deoxycytidine (5-AZA-CdR)	Upregulate endogenous retrovirus (ERV) with G9Ai, synergistically induce antitumor	[158]
5-Azacytidine (5AZA-C)	Induced the recruitment of activated (IFNγ^+^) CD4^+^T cells, CD8^+^ T cells and NK cells combining with α-difluoromethylornithine (DFMO)	[159]
HDACi		
Entinostat (class I HDACi)	Synergistic effect with cisplatin in HGSOC	[160]
Vorinostat	Inhibit tumor growth and prolong survival via targeting CD146	[161]
Panobinostat	1. Synergistic effect with carboplatin in EOC	[162]
	2. Reduce homologous recombination (HR) cyclin E-overexpression, as a means of enhancing PARPi activity	[163]
Thailandepsins	Inhibit cell viability and induce apoptosis	[164]
Suberoylanilide hydroxamic acid (SAHA)	Combined olaparib induced apoptosis and pH2AX expression more strongly to a greater extent than either drug alone	[165]

DNMTis have been used for cancer immunotherapy, and representative nucleic acid analogs such as decitabine (DAC) and azacitidine (AZA). DNMTis have been approved for the treatment of acute myeloid leukemia (AML), chronic myelomonocytic leukemia (CMML), and myelodysplastic syndromes (MDS) and have been widely used in immunotherapeutic clinical trials of multiple cancers [114]. In OC cell lines, DNMTi treatment upregulates the expression of the antigen-processing and presentation molecules B2M, CALR, CD58, PSMB8, and PSMB9, demonstrating a possible mechanism for sensitizing ovarian tumors to immunotherapy [115]. By reducing the mRNA and protein levels of DNA methylase, ginsenoside Rg3 can promote the antitumor effects of *p53*, *p16*, and *hMLH-1* in OC cells, inhibiting the migration and invasion of cancer cells, and promoting cell apoptosis [116]. In HGSOC, 5-hydroxymethylcytosine (5-hmC) loss is an epigenetic hallmark that is associated with a poor overall survival rate, shorter time to relapse, and a reduced response to platinum-based chemotherapy [117]. DNMTi pretreatment restores 5-HMC loss and sensitivity to platinum chemotherapy. Recently, the DNMTi guadecitabine in combination with the PARPi talazoparib has been shown to increase the sensitivity of OC cells to PARPis, independent of the BRCA status [118]. The development of resistance and severe side effects are current therapeutic challenges for DNMTis that need to be overcome, and the hypermethylation of CpGs may be a novel mechanism of action for DNMTis. This finding provides a new idea for predicting the therapeutic efficacy and side effects of DNMTis [119].

HDACis are another promising new class of anticancer drugs that can induce cancer cell cycle arrest, differentiation, and cell death; reduce angiogenesis; and regulate the immune response [120]. Among the currently available  HDACis, four have been tested in OC, including vorinostat, romidepsin, valproate, and PXD101. *PAX8* is an EOC proto-oncogene. HDACis interfere with the transcription of *PAX8* and downstream factors by blocking the acetylation of histone H3K27 [121]. IKK inhibitors can improve the efficacy of HDACis in OC and that of other solid tumors by inhibiting IL-8 [122]. DHRS2 expression is decreased in OC, and high DHRS2 expression is correlated with a better prognosis. HDACis increase the mRNA and protein levels of DHRS2, suggesting that HDACis improve the prognosis of OC patients by upregulating the expression of DHRS2 [123]. As a single drug, HDACis effectively inhibit the growth and spread of ovarian tumors and synergize with platinum-based chemotherapy drugs, which shows a real potential for clinical success [124]. The efficacy of HDACis in the treatment of solid tumors remains uncertain. A hybrid HDAC inhibitor, the hybrid molecule Roxyl-ZHC-84, has been developed. It greatly improves the limitations of traditional HDAC inhibitors in solid tumors by overcoming JAK1-STAT3-BCL2-mediated drug resistance and provides new ideas for the further research and development of antitumor drugs [125].

DNMTi and HDACi can increase the antitumor immunogenicity of cancer cells with beneficial effects on the immune microenvironment of ovarian tumors [126]. The combination of DNMTis and HDACis for the treatment of elderly patients with AML has been approved [127]. Dual inhibition may be a novel epigenetic therapy combination that can be used as a novel strategy for the treatment of OC.

## Conclusions

Epigenetics is likely involved in the origin and progression of OC and will likely be an important treatment adjunct for OC. Epigenetics will likely provide an important tool for early molecular cancer screening and predictive markers for the selection of drug treatment protocols for high-risk patients. To date, most studies are in an early stage, and more intensive investigation is required. There is a limited understanding of OC disease progression; hence, the useful clinical application of epigenetics requires further investigation.

OC has an insidious onset, with multiple histological subtypes and complex molecular expression patterns. There are limitations to existing investigations in that reported study sample sizes are small, and the association of miRNA with disease occurrence lacks a causal relationship, with no identified specific OC biomarkers that can provide a direction for the analysis of miRNA. Furthermore, the lack of standardized protocols for sample collection and RNA extraction, as well as the less than ideal selection of individual patient differences, makes it difficult to compare reported results. LncRNAs are involved in the origin, invasion, and metastasis of OC. LncRNA function is multifaceted, with an array of complex cellular and molecular activities. Some lncRNAs show almost ubiquitous effects in OC, and it will be interesting to consider whether most of them have specific functions and may influence the extent to which lncRNAs play a role in cancer. Abnormal DNA methylation and histone modification directly affect tumor progression and drug tolerance. DNA methylation is often used to compare the methylation status of specific genes in normal and OC cells. However, analysis of the genome-wide DNA methylation status is limited in that there is wide variability in sample size, tissue type, and analysis methodology. The analysis of histone-modified proteins remains in an early stage, and clinical trials of inhibitors are underway.

Understanding the molecular mechanisms underlying chemotherapeutic resistance is critical to treatment decisions and to the discovery of new anticancer drug targets. DNMTis and HDACis, or even a combination of the two, show great potential for the targeted treatment of OC. Currently, DNMTis are being evaluated in both preclinical and clinical settings, although cytotoxic side effects currently limit the clinical application of demethylating drugs. With the development of large-scale genome projects and sequencing technologies, such as the Encyclopaedia of DNA Elements (ENCODE) [128] and TCGA Project [129], our understanding of the mechanistic basis for treatment responses will become more important. Understanding the tumor tissue type, the gene sequence of an individual tumor, and the immune tumor microenvironment will allow the use of epigenetic drugs, immune regulators, targeted therapies, or a combination of these therapies to improve the clinical management of OC.

In the future, more research is needed to validate the biological mechanisms and clinical implications of tumor characterization at the molecular level, putting them into clinical practice. In addition, non-invasive approaches, such as diagnostic biomarkers in the blood or urine, are superior to invasive biopsy procedures. Immunotoxicity and other reactions will be important considerations when using epigenetic-based therapeutics. Thus, epigenetic studies have already added an additional layer of complexity to the understanding of OC, although the mechanistic understanding of biological functions is only beginning to develop. The lack of appropriate detection systems and therapeutic targets for OC are still major challenges. The development of epigenetics has opened a new horizon to discover specific biomarkers and therapeutics that could ultimately change the future of OC diagnosis and treatment.

## Data Availability

Not applicable.

## Abbreviations

OC: Ovarian cancer
EOC: Epithelial ovarian cancer
OSE: Ovarian surface epithelium
HGSOC: High-grade serous ovarian cancer
ncRNA: Non-coding RNA
miRNA: MicroRNA
lncRNA: Long non-coding RNA
DNMTi: DNA methylation inhibitor
HDACi: Histone deacetylase inhibitor
snRNA: Small nuclear RNA
snoRNA: Small nucleolar RNA
RNAi: RNA interference
sncRNA: Small non-coding RNA
siRNA: Short interfering RNA
bp: Base pairs
NF-κB: Nuclear factor kappa B
DNMTs: DNA methyltransferases
CpG: Cytosine-phosphate-guanine
HAT: Histone acetyltransferase
HDAC: Histone deacetylase
EMT: Epithelial–mesenchymal transition
LRRK2: Leucin-rich repeat kinase 2
DFS: Disease-free survival
OS: Overall survival
TCGA: The Cancer Genome Atlas
GO: Gene Ontology
GSEA: Gene Set Enrichment Analysis
HOTAIR: HOX antisense intergenic RNA
MALAT1: Metastatic specific lung adenocarcinoma transcript 1
MMP3: Matrix metalloproteinase 3
IGF2: Insulin-like growth factor 2
NEAT1: Nuclear paraspeckle assembly transcript 1
LSINCT5: Long stress-induced non-coding transcript 5
CCAT2: Colon cancer-associated transcript 2
CERNA2: Competing endogenous lncRNA 2
UCA1: Urothelial cancer-associated 1
BC200: Brain cytoplasmic RNA 200
GAS5: Growth arrest-specific 5
IFN: Interferon
PARPi: PARP inhibitor
DAC: Decitabine
AZA: Azacitidine
AML: Acute myeloid leukemia
CMML: Chronic myelomonocytic leukemia
MDS: Myelodysplastic syndrome
5-AZA-CdR: 5-Aza-2′-deoxycytidine
ERV: Endogenous retrovirus
5AZA-C: 5-Azacytidine
DFMO: Difluoromethylornithine
HR: Homologous recombination
SAHA: Suberoylanilide hydroxamic acid
H3K27me3: Histone H3 lysine 27 trimethylation
ATRA: All-trans retinoic acid
TERT: Telomerase reverse transcriptase
USP1: Ubiquitin-specific protease 1
ENCODE: Encyclopaedia of DNA Elements
